# Re-Treatment Tuberculosis Cases Categorised as “Other”: Are They Properly Managed?

**DOI:** 10.1371/journal.pone.0028034

**Published:** 2011-12-14

**Authors:** Hannock Tweya, Henry Kanyerere, Anne Ben-Smith, John Kwanjana, Andreas Jahn, Caryl Feldacker, Dickman Gareta, Limbani Mbetewa, Mathew Kagoli, Mike Tikhalenawo Kalulu, Ralf Weigel, Sam Phiri, Mary Edginton

**Affiliations:** 1 The International Union Against Tuberculosis and Lung Disease, Paris, France; 2 Lighthouse Trust, Lilongwe, Malawi; 3 The National Tuberculosis Control Programme, Ministry of Health, Lilongwe, Malawi; 4 Maame Akua, Lilongwe, Malawi; 5 Department for HIV and AIDS, Centre for Monitoring and Evaluation Department, Ministry of Health, Lilongwe, Malawi; 6 International Training and Education Center for Health, University of Washington, Seattle, Washington, United States of America; San Francisco General Hospital, University of California San Francisco, United States of America

## Abstract

**Background:**

Although the World Health Organization (WHO) provides information on the number of TB patients categorised as “other”, there is limited information on treatment regimens or treatment outcomes for “other”. Such information is important, as inappropriate treatment can lead to patients remaining infectious and becoming a potential source of drug resistance. Therefore, using a cohort of TB patients from a large registration centre in Lilongwe, Malawi, our study determined the proportion of all TB re-treatment patients who were registered as “other”, and described their characteristics and treatment outcomes.

**Methods:**

This retrospective observational study used routine program data to determine the proportion of all TB re-treatment patients who were registered as “other” and describe their characteristics and treatment outcomes between January 2006 and December 2008.

**Results:**

1,384 (12%) of 11,663 TB cases were registered as re-treatment cases. Of these, 898 (65%) were categorised as “other”: 707 (79%) had sputum smear-negative pulmonary TB and 191 (21%) had extra pulmonary TB. Compared to the smear-positive relapse, re-treatment after default (RAD) and failure cases, smear-negative “other” cases were older than 34 years and less likely to have their HIV status ascertained. Among those with known HIV status, “other” TB cases were more likely to be HIV positive. Of TB patients categorised as “other”, 462 (51%) were managed on the first-line regimen with a treatment success rate of 63%.

**Conclusion:**

A large proportion of re-treatment patients were categorised as “other”. Many of these patients were HIV-infected and over half were treated with a first-line regimen, contrary to national guidelines. Treatment success was low. More attention to recording, diagnosis and management of these patients is warranted as incorrect treatment regimen and poor outcomes could lead to the development of drug resistant forms of TB.

## Introduction

With an annual incidence of 260 tuberculosis (TB) cases per 100,000 population [1], TB continues to threaten the lives of many people in Malawi. In the last 10 years, the number of previously-treated TB cases increased from 5% to 11% (Malawi National TB Programme,, unpublished data). This is of concern as these patients may acquire drug resistance and, therefore, have poor treatment outcomes [2] [3]. The Malawi National TB Programme (NTP) diagnoses, registers and treats all forms of TB following international guidelines [4] [5]. Previously-treated TB patients are classified into four categories: relapse, failure, re-treatment after default (RAD) and “other”. According to Malawi National TB guidelines, “other” includes chronic re-treatment TB patients, patients with recurrent smear-negative pulmonary TB and extra-pulmonary TB [4].

The categorization of TB patients as “other” may indicate poor patient management. According to the World Health Organization's (WHO) Global Tuberculosis Control report on relapse, failure, RAD and “other” for Africa, South-East Asia and Western Pacific regions in 2007, about 35% of 581,000 TB re-treatment cases were registered as “other” [1]. Although the WHO report provides information on the number of patients categorised as “other”, there are limited data on treatment regimens or treatment outcomes for this group. A study conducted in India found that TB patients categorized as “other” were treated with a category II regimen and had better treatment outcomes compared to relapse, failure and RAD [6]. In a routine programme setting, information on treatment regimen and outcomes is important as inappropriate management of TB patients can lead to patients remaining infectious and becoming a potential source of drug resistance [7].

We therefore carried out a study at the largest TB registration centre in Malawi to determine the numbers and proportions of adult TB re-treatment patients categorised as “other”. We also documented what treatment regimens were used and patient treatment outcomes for “other” TB patients, comparing them to outcomes reported for previously-treated patients registered as relapse, failure and RAD.

## Methods

### Setting

The study was conducted at Martin Preuss Centre (MPC) clinic in Lilongwe, the largest TB registration centre in Malawi. MPC registers 4,000 TB patients annually. Diagnosis of PTB is based on clinical examination, sputum smear microscopy, and chest radiography. In most cases, diagnosis of EPTB is made based on radiography but it was also diagnosed bacteriogically or histopathologically. For quality control, peripheral laboratories send standard smears with known results to the central laboratory at MPC every month; these smears are rechecked by central laboratory technicians. Once diagnosed with TB, patients are registered by the district TB officer and initiated on standardized treatment regimens [4]. According to national guidelines, all re-treatment patients should be treated with the standard WHO category II regimen, consisting of two months of daily streptomycin (S), rifampicin (R), Isoniazid (H), pyrazinamide (Z) and ethambutol (E), followed by one month of RHZE and then five months of RHE (2SRHZE/1RHZE/5RHE).

Approximately 30% of patients initiated and completed their treatment at MPC; 70% initiated at MPC but chose to complete their treatment at one of 18 peripheral health facilities. Treatment cards for those who completed treatment at MPC remained with the TB officer on site; treatment cards of patients who sought care at peripheral health facilities were sent to, and maintained, at their respective facilities during treatment. After completion of treatment, the cards from peripheral sites should be returned to the initial registration centre (MPC). Every six months, the district TB officer visited peripheral health facilities to collect treatment cards that were not returned. Treatment outcomes (“cured”, “treatment completed”, “died”, “treatment failure”, “defaulted” or “transferred-out”) were updated in the TB Register at MPC from treatment cards.

### Study design and participants

This retrospective cohort study of re-treatment adult TB patients (age≥15 years) was conducted using routine program data from TB registers and patient treatment cards collected from January 2006 to December 2008 at MPC. The Malawi National TB control program classifies previously treated patients as follows: 1) “relapse” = completed treatment but subsequently developed active smear-positive pulmonary TB; 2) “failure” = remained sputum smear-positive at five months or more during first-line treatment; 3) “re-treatment after default (RAD)” = interrupted treatment for more than two months and returned with smear-positive TB; and 4) “other” = not fulfilling any of the above categories, including chronic cases [4].

### Data collection and Data Analysis

Data extracted from registers for each patient included TB registration number, registration date, age, gender, TB classification, patient category, treatment regimen, initial sputum microscopy results and HIV status. The Malawi TB programme does not routinely conduct culture tests and, as such, there were no culture results captured in the national TB register. HIV status was only documented from January 2007. Treatment outcomes were extracted from treatment cards. Missing case registration data in the TB registers were retrieved from treatment cards, if available.

Data were double-entered and cleaned in a custom-designed MS Access database and analyzed in STATA 10. Since there was a strong correlation between TB classification and patient retreatment category, only patient category was considered for further analysis. We categorized re-treatment patients in four ways: smear-positive relapse, smear-positive RAD, smear-positive failure and smear-negative “other”. The “other” re-treatment category consisted of smear-negative PTB and EPTB patients except for 3 cases that were registered in 2006. These cases might have been misclassified and were therefore excluded from further analyses. The chi-square test for significance was used to compare patient characteristics and patient re-treatment categories. Because TB treatment outcomes are generally classified as either treatment success or not, we modelled treatment outcome as a binary variable of success versus all other treatment outcomes. Logistic regression was then used to identify patient characteristics that were independently associated with treatment success. The final multivariable model was determined using forward selection, including explanatory variables with a two-sided p-value of ≤0.05. Age was included *a priori* in all multivariable models. The log-likelihood ratio test was used to assess the independent contribution of explanatory variables to the models. Statistical significance was determined at the *p*≤0.05 level, shown with 95% confidence intervals (CI) throughout.

### Ethics Approval

The study was approved by the Malawi National Health Science Research and the Ethics Advisory Group of The International Union Against Tuberculosis and Lung Disease, Paris, France. The ethics committees waived the need for patient consent because the study used routine programmatic data that did not include any personal identifiers and were analysed anonymously.

## Results

### Characteristics of re-treatment versus new cases

Between January 2006 and December 2008, 11,663 adult TB cases were registered at MPC. Case registration data were available for all TB patients. Of all TB registrations, 11,653 (99.9%) had smear microscopy: 3,094 (27%) had positive smear results. A total of 1,384 (12%) were registered as ‘re-treatment cases’. Compared to new TB cases, re-treatment cases were more likely to be female (60% vs. 56%, p = 0.04), older than 34 years (55% vs. 45%, p<0.001), and less likely to have known HIV status (58% vs. 63%, p<0.001) (**Table 1**). Among those with known HIV status, re-treatment cases were more likely to be HIV-positive (71% vs. 66%, p = 0.006).

**Table 1:** Distribution of patient characteristics by treatment category (n = 11,663 cases).

Characteristics	New cases		Re-treatment		*p*-value
	n	%	n	%	
**Sex**					
Female	4,427	43%	556	40%	*0.041*
Male	5,852	57%	828	60%	
**Age**					
15–34	5,558	54%	620	45%	*<0.001*
≥35	4,672	45%	764	55%	
Unknown	49	1%	0	0%	
**HIV status**					
Positive	4,309	42%	575	41%	*<0.001*
Negative	2,182	21%	233	17%	
Unknown	3,788	37%	576	42%	
**Smear status** ¥					
Positive	2,606	25%	486	35%	*<0.001*
Negative	7,671	75%	898	65%	
**Year of registration**					
2006	3,295	86%	531	14%	*<0.001*
2007	3,279	89%	426	11%	
2008	3,705	90%	430	10%	

¥ 2 cases had unknown smear status.

### Characteristics of smear-negative “other” versus smear-positive relapse, RAD and failure cases

Of 1,384 re-treatment cases, 898 (65%) were classified as smear-negative “other”; 406 (29%) as smear-positive relapse; 46 (4%) as smear-positive RAD; and 34 (3%) as smear-positive failure. Compared to the smear-positive relapse, RAD and failure cases, smear-negative “other” cases were older than 34 years and less likely to have their HIV status ascertained. Among those with known HIV status, “other” TB cases were more likely to be HIV-positive (**Table 2**).

**Table 2:** Distribution of patient characteristics by re-treatment category (n = 1,384).

Characteristics	Re-treatment									*P-value* ¥
	Total	Smear-positive Relapse		Smear-positive RAD		Smear-positive Failure		Smear-negative Other		
**Sex**										
Male	**828**	250	*62%*	*35*	*76%*	*22*	*65%*	521	*58%*	*0.068*
Female	**556**	156	*38%*	*11*	*24%*	*12*	*35%*	377	*42%*	
**Age**										
15–34	**620**	206	*51%*	*33*	*71%*	*23*	*68%*	*358*	*40%*	*0.001*
≥35	**764**	200	*49%*	*13*	*28%*	*11*	*32%*	*540*	*60%*	
**HIV status**										
Positive	**575**	163	*40%*	21	*46%*	12	*35%*	379	*42%*	*0.001*
Negative	**233**	78	*19%*	16	*35%*	11	*32%*	128	*14%*	
Unknown	**576**	165	*41%*	9	*20%*	11	*32%*	391	*43%*	
**Treatment regimen**										
First line: 2RHZE/4RH	**500**	28	7%	7	*15%*	3	*9%*	462	*51%*	*<0.001*
Re-treatment: 2SRHZE/1RHZE/RHE	**754**	348	86%	34	*74%*	30	*88%*	342	*38%*	
Unknown	**130**	30	7%	5	*11%*	1	*3%*	94	*10%*	
**Year of registration**										
2006	**528**	145	*36%*	7	*15%*	11	*32%*	365	*41%*	*<0.001*
2007	**426**	139	*34%*	11	*24%*	16	*47%*	260	*29%*	
2008	**430**	122	*30%*	28	*61%*	7	*21%*	273	*30%*	

¥ Chi-square test for the differences in the distribution of the categorical characteristic across patient re-treatment categories.

### Treatment management

Of the 898 smear-negative “other” TB cases, 342 (38%) received the category II re-treatment TB drug regimen while 462 (51%) were inappropriately managed on first-line treatment regimen (2RHZE/4RH). Information on treatment regimen was missing in 94 (10%) patients. A total of 348 (86%) relapse cases, 34(74%) RAD and 30 (88%) failure cases received the category II regimens, indicating that significantly more smear-positive retreatment cases received a category II regimen (p<0.001).

### Treatment outcomes

Treatment outcomes were recorded for 663 (48%) of 1,384 re-treatment cases. Of the 663, 585 (88%) had PTB and 78 (12%) had EPTB. Treatment cards were missing for 721 (52%); these patients were not included in further analysis. According to the register, re-treatment patients with treatment cards were similar to those without treatment cards in terms of age (p = 0.194), sex (p = 0.557) TB classification (p = 0.092) and place of TB treatment (p = 0.070). Of all outcomes, 66% were treatment success, 20% loss to follow-up, 11% death, 2% transfer-out and 1% treatment failure. Univariable analysis of re-treatment patients with treatment cards showed that sex (p = 0.003), treatment regimen (p = 0.033) and re-treatment category (p = 0.050) were significantly associated with treatment outcome; HIV status (p = 0.683), age (p = 0.248) and year of registration (p = 0.659) were not (**Table 3**).

**Table 3:** Distribution of patient characteristics by treatment outcomes among re-treatment TB patients with treatment cards (n = 663).

Characteristics	Treatment Outcome					*P-value* ¥
	n	Successful		Not successful		
**Total**	**663**	***435***		**228**		
**Sex**						
Male	**402**	246	*57%*	156	*68%*	*0.003*
Female	**261**	189	*43%*	72	*32%*	
**Age**						
15–34	**285**	180	*41%*	105	*46%*	*0.248*
≥35	**378**	255	*59%*	123	*54%*	
**HIV status**						
Positive	**309**	204	*47%*	105	*46%*	*0.683*
Negative	**146**	99	*23%*	47	*21%*	
Unknown	**208**	132	*30%*	76	*33%*	
**Re-treatment category**						
Smear-positive Relapse	**204**	149	*34%*	55	*24%*	*0.050*
Smear-positive RAD	**30**	17	*4%*	13	*6%*	
Smear-positive Failure	**21**	12	*2%*	9	*4%*	
Smear-negative “other” (EPTB, PTB)	**407**	257	*59%*	150	*66%*	
**Treatment regimen**						
First line: 2RHZE/4RH	**214**	144	*67%*	70	*33%*	*<0.033*
Re-treatment: 2SRHZE/ 1RHZE/RHE	**411**	274	*67%*	137	*33%*	
Unknown	**37**	17	*46%*	20	*54%*	
**Year of registration**						
2006	**181**	118	*65%*	63	*35%*	*0.659*
2007	**189**	120	*63%*	69	*37%*	
2008	**292**	197	*67%*	95	*33%*	

¥ Chi-square test,

Results from logistic regression (**Table 4**) indicated that females were more likely to have better treatment outcomes than males (OR = 1.68, 95% CI 1.20–2.37). Treatment success was also significantly higher in smear-positive relapse patients than in the smear-negative “other” (OR = 1.58, 95% CI 1.09–2.28). There was no significant difference in treatment success between smear-positive RAD patients (57%, OR = 0.76 CI 0.36–1.62) or smear-positive failure (57%, OR = 0.77 0.32–1.89) compared to smear-negative “other”. Multivariable analysis showed no significant associated between treatment success and treatment regimen. However, females had a higher probability of having good treatment outcomes compared to males (OR = 1.71, 95% CI 1.21–2.41). Treatment success rates remained higher among smear-positive relapse compared with the smear-negative “other” (adjusted OR = 1.63, 95% CI 1.09–2.45). Treatment success rates were similar between the smear-negative “other” and smear-positive RAD or smear-positive failure cases (smear-positive RAD adjusted OR = 0.86 95% CI 0.39–1.86; smear-positive failure OR = 0.85, 0.34–2.13). Re-treatment TB category did not modify the association between treatment regimen and treatment outcomes.

**Table 4:** Factors associated with binary treatment success rate among TB treatment classification (n = 663).

Characteristics	N	Unadjusted Odds Ratio (95% CI)	*P-value* *	AdjustedF- Odds Ratio (95% CI)	*P-value* *
**Sex**					
Female	261	1.68 (1.20–2.37)	*0.004*	1.71 (1.21–2.41)	*0.002*
Male	402	1.00		1.00	
**Age**					
15–34	285	0.82 (0.59–1.13)	*0.229*	0.79 (0.55–1.11)	*0.170*
≥35	378	1.00		1.00	
**Re-treatment category**					
Smear-positive Relapse	204	1.58 (1.09–2.28)	*0.047*	1.63 (1.09–2.45)	*0.065*
Smear-positive RAD	30	0.76 (0.36–1.62)		0.86 (0.39–1.86)	
Smear-positive Failure	21	0.77 (0.32–1.89)		0.85 (0.34–2.13)	
Smear-negative “other”(EPTB, PTB)	407	1.00		1.00	
**Treatment regimen**					
First line: 2RHZE/4RH	214	1.02 (0.72–1.46)	*0.040*	1.16 (0.79–1.71)	*0.078*
Re-treatment: 2SRHZE/1RHZE/5RHE	411	1.00		1.00	
Unknown	37	0.43 (0.22–0.84)		0.50 (0.25–1.02)	

* P-value for likelihood ratio test,

^F-^Adjusted for sex, age and patient category, RAD = Re-treatment after default.

## Discussion

This is the first study from a routine TB programme setting in sub-Saharan Africa to explore characteristics and treatment outcomes in previously-treated adult TB patients registered as “other”. In this setting, 65% of the re-treatment cases were classified as “other”. Among “other”, 75% had smear-negative PTB, 75% were HIV-positive and most were men older than 34 years. The study also showed a high proportion of all TB cases had smear microscopy results, indicating the TB programme performed well. According to Malawi national TB guidelines, a re-treatment regimen (2SRHZE/1RHZE/5RHE) should have been used for all re-treatment cases. More than half of the patients classified as “other” were inappropriately treated with the first-line TB regimen (2RHZE/4RH). However, overall treatment success rate was low among re-treatment TB patients receiving a category II regimen, suggesting that this regimen may also not be the most appropriate.

There are several possible explanations for the large number of “other” patients in this study. Many of these patients had smear-negative TB and were HIV-positive, raising questions about whether these were truly recurrent smear-negative PTB as a result of re-infection or reactivation [8], or whether they had HIV-related disease that was misdiagnosed as TB [9] [10]. It is also possible that these patients had other pulmonary or cardiac disease that was misdiagnosed as TB [11]. Similar misdiagnosis might have also occurred among EPTB cases because of their atypical presentations. A previous study conducted in Malawi found that 16% of patients with an EPTB diagnoses did not have TB [12]. Adoption of new accurate diagnostic tools such as GeneXpert MTB/RIF might help to reduce misdiagnosis in this group of patients. We also noted uncertainty among some TB clinical officers in other TB clinics as to how the “other” category should be defined. Interestingly, WHO TB treatment guidelines considers the “other” category as a variable group: “other” describes patients who returned with smear-negative PTB or EPTB, previously treated but with unknown outcome, or unknown whether they were ever treated or not [13]. With such a sizeable and heterogeneous group of “other” cases, more effort is needed to classify these cases into clear, exhaustive, mutually-exclusive categories. It may be worthwhile creating additional disease categories from the “other” group such as “presumed re-treatment TB” for patients with unknown previous treatment status; “smear-negative re-treatment TB” for patients who return with smear-negative PTB or EPTB; and “other re-treatment” for previously-treated patients with unknown outcomes. Overall, better diagnostic algorithms, improved access to mycobacterial culture facilities or rapid and accurate TB diagnostic tests are needed to reduce misdiagnosis and poor management.

Similar to other studies [14], [15], we also observed low treatment success rate among re-treatment cases. The most important reasons for low treatment success rates were loss to follow-up (20%) and death (11%). The high proportion of patients lost to follow-up might indicate a need for closer follow-up of this group during TB treatment as loss to follow up may include hidden deaths. Also, despite the overall low treatment outcome rates, the treatment success rate for smear-positive relapse was significantly higher than the treatment success rate in “other” TB patients after adjusting for sex and age. There are various possible explanations. Relapse patients have microbiologically-proven TB in contrast to “other” cases, which, as discussed above, may be a hybrid of true TB, HIV-related disease and other pulmonary or cardiac disease. Given high HIV infection rates in “other” cases and the inclusion of conditions that may not respond to TB treatment, only a moderate proportion might improve clinically (possibly due to other concomitant management). This is consistent with the 63% TB treatment success rate in the “other” group, and the inappropriate use of first-line TB regimen would not have adversely affected clinical outcomes in such patients. It is also likely that many HIV-infected smear-negative “other” TB category patients were more immune-compromised than relapse smear-positive PTB patients, resulting in poorer outcomes [16]. As this study was retrospective, we have no means of further investigating these issues. Generally, the re-treatment group had low re-treatment failure rates which may indicate a low prevalence of MDR-TB, although this does require more in depth study. Our results also showed that males had lower treatment success rates and were more likely to default or die while on TB treatment, and this may reflect low compliance with TB treatment therapy [17], [18].

Although neither the WHO nor The International Union Against Tuberculosis and Lung Disease TB treatment guidelines provide clear guidance on what regimen should be given to patients classified as “other”, the current Malawi National TB treatment guidelines recommend re-treatment drug regimens for all “other” TB patients. However, less than half of “other” TB patients in our study were treated with a category II regimen, suggesting clinician's uncertainty about this category of TB patients, rather than a general failure to follow guidelines. Other countries treat “other” TB patients with a first-line regimen if their smear is negative because they are considered to be at lower risk of failure and developing resistance. However, some previous studies observed generally low treatment success rates among smear positive re-treatment TB patients treated with the category II regimen [15], [19]. Given the poor treatment outcomes also observed in these “other” re-treatment groups, it is certainly worthwhile and important to review the current category II regimen for re-treatment TB patients. These patients may be at high risk of drug resistance and of developing MDR, as was seen in South Africa [20].

Our findings should be viewed with the following limitations. First, 52% of treatment cards for re-treatment patients were missing although all treatment cards should have been returned to the MPC TB registration centre at the time of this study. Some cards might have been lost at the peripheral sites or within the TB registry, introducing bias if patients without treatment cards had different outcomes than those with treatment cards. However, we observed no differences in terms of demographics, TB classification and place of treatment between patients with and without treatment cards. Second, although TB outcome data were supposed to be recorded on treatment cards and in the TB register, a considerable proportion of adverse outcomes were not recorded in the TB registers. As treatment cards are the primary source of outcome information, we based the analysis on outcomes documented on the cards and not the register. Exclusion of patients with missing cards may have resulted in a biased selection of patients with better treatment outcomes, assuming that cards of defaulters were more likely to be lost. Despite these limitations, the study findings are useful to inform policy and programs in Malawi and other comparable settings to improve care for TB patients.

In conclusion, our study shows that the “other” TB category constitutes a sizeable population of re-treatment patients and is poorly managed. Accurate definitions and categories from the current “other” category should be created to avoid poor patient management. The high proportion of missing treatment cards suggests that less priority is given to these re-treatment patients. Treatment success may be improved by adoption of more accurate diagnostic tools such as Xpert MTB/RIF and more rigorous training and regular supervision of TB staff. There is also a need to reappraise the category II regimen as our results suggest that use of this regimen is not associated with successful outcomes. Some of these issues are currently under discussion within the Malawi NTP with the aim of improving routine programme management.
